# MAB-MIG: registry of the spanish neurological society of erenumab for migraine prevention

**DOI:** 10.1186/s10194-021-01267-x

**Published:** 2021-07-17

**Authors:** Robert Belvís, Pablo Irimia, Patricia Pozo-Rosich, Carmen González-Oria, Antonio Cano, Javier Viguera, Belén Sánchez, Francisco Molina, Isabel Beltrán, Agustín Oterino, Elisa Cuadrado, Angel Gómez-Camello, Miguel Alberte-Woodward, Carmen Jurado, Teresa Oms, David Ezpeleta, Javier Díaz de Terán, Noemí Morollón, Germán Latorre, Marta Torres-Ferrús, Alicia Alpuente, Raquel Lamas, Carlos Toledano, Rogelio Leira, Sonia Santos, Margarita Sánchez del Río

**Affiliations:** 1 Headache and Neuralgia Unit, Department of Neurology, Hospital de La Santa Creu I Sant Pau, C/ Mas Casanova 90, CP08025 Barcelona, Spain; 2 Clínica Universitaria de Navarra, Pamplona, Spain; 3 Headache Unit, Neurology Department, Hospital Universitari Vall D´Hebron, Barcelona, Spain; 4 Headache and Neurological Pain Research Group, Vall D´Hebron Pain Research Group, Vall D´Hebron Research Institute, Department of Medicine, Universitat Autónoma de Barcelona, Barcelona, Spain; 5 Hospital Universitario Virgen del Rocío, Sevilla, Spain; 6 Hospital de Mataró, Barcelona, Spain; 7 Hospital Universitario Virgen de La Macarena, Sevilla, Spain; 8 Hospital Quironsalud, Zaragoza, Spain; 9 Hospital Son Espases, Palma de Mallorca, Spain; 10 Hospital General Universitario de Alicante, Alicante, Spain; 11 Hospital Universitario Marqués de Valdecilla, Santander, Spain; 12 Hospital del Mar, Barcelona, Spain; 13 Hospital Universitario San Cecilio, Granada, Spain; 14 Hospital Universitario Lucus Augusti, Vigo, Spain; 15 Hospital Universitario Reina Sofía, Córdova, Spain; 16 Hospital Dos de Maig, Barcelona, Spain; 17 Hospital Quironsalud, Madrid, Spain; 18 Hospital Universitario La Paz, Madrid, Spain; 19 Hospital Universitario Dexeus, Barcelona, Spain; 20 Hospital Universitario de Fuenlabrada, Madrid, Spain; 21 Hospital Universitario de Santiago de Compostela, de Compostela, Spain; 22 Hospital Clínico Universitario Lozano Blesa, Zaragoza, Spain; 23 Clínica Universitaria de Navarra, Madrid, Spain

**Keywords:** Erenumab, Migraine, Monoclonal antibody, Preventive treatment, Registry

## Abstract

**Background:**

Erenumab was approved in Europe for migraine prevention in patients with ≥ 4 monthly migraine days (MMDs). In Spain, Novartis started a personalized managed access program, which allowed free access to erenumab before official reimbursement. The Spanish Neurological Society started a prospective registry to evaluate real-world effectiveness and tolerability, and all Spanish headache experts were invited to participate. We present their first results.

**Methods:**

Patients fulfilled the ICHD-3 criteria for migraine and had ≥ 4 MMDs. Sociodemographic and clinical data were registered as well as MMDs, monthly headache days, MHDs, prior and concomitant preventive treatment, medication overuse headache (MOH), migraine evolution, adverse events, and patient-reported outcomes (PROs): headache impact test (HIT-6), migraine disability assessment questionnaire (MIDAS), and patient global improvement change (PGIC). A > 50% reduction of MMDs after 12 weeks was considered as a response.

**Results:**

We included 210 patients (female 86.7%, mean age 46.4 years old) from 22 Spanish hospitals from February 2019 to June 2020. Most patients (89.5%) suffered from chronic migraine with a mean evolution of 8.6 years. MOH was present in 70% of patients, and 17.1% had migraine with aura. Patients had failed a mean of 7.8 preventive treatments at baseline (botulinum toxin type A—BoNT/A—had been used by 95.2% of patients). Most patients (67.6%) started with erenumab 70 mg. Sixty-one percent of patients were also simultaneously taking oral preventive drugs and 27.6% were getting simultaneous BoNT/A. Responder rate was 37.1% and the mean reduction of MMDs and MHDs was -6.28 and -8.6, respectively. Changes in PROs were: MIDAS: -35 points, HIT-6: -11.6 points, PIGC: 4.7 points. Predictors of good response were prior HIT-6 score < 80 points (*p* = 0.01), ≤ 5 prior preventive treatment failures (*p* = 0.026), absence of MOH (*p* = 0.039), and simultaneous BoNT/A treatment (*p* < 0.001). Twenty percent of patients had an adverse event, but only two of them were severe (0.9%), which led to treatment discontinuation. Mild constipation was the most frequent adverse event (8.1%).

**Conclusions:**

In real-life, in a personalized managed access program, erenumab shows a good effectiveness profile and an excellent tolerability in migraine prevention in our cohort of refractory patients.

## Background

Migraine is the second leading neurological cause of disability and the first among young women according to the GBD2019 [1]; and approximately 38% of migraine patients [2] need a preventive treatment to reduce this disability. In Spain, several first line preventive drugs for episodic migraine (EM) are available: topiramate, sodium valproate, amitriptyline, flunarizine and beta-blockers, but only botulinum toxin type A (BoNT/A) and topiramate are available for chronic migraine (CM) [3].

The number of monthly migraine days (MMDs) after 12 weeks of treatment is the main variable of efficacy for a preventive drug in migraine [4], despite the known decrease in prevention adherence beyond 12 weeks [5–7]. The loss of effectiveness and side effects account for this progressive reduction in adherence. For these reasons, we urgently needed new preventive drugs, and anti-CGRP monoclonal antibodies (CGRP mAbs) have been developed to cover these needs.

CGRP is a neuropeptide distributed throughout the human body and highly concentrated in the trigeminovascular system [8]. The levels of CGRP are increased during the migraine attack in blood, tears, saliva, and cerebrospinal fluid, and normalized after the attack [9, 10]. They are also permanently increased during CM [11]. Moreover, the intravenous administration of CGRP causes migraine-like headaches in migraine patients and voluntaries [12]. Therefore, CGRP is an excellent target for migraine therapy.

At present, there are three subcutaneous CGRP mAbs marketed in Spain: erenumab (Aimovig®), galcanezumab (Emgality®) and fremanezumab (Ajovy®). CGRP mAbs block the CGRP receptor (erenumab) or the ligand itself (galcanezumab and fremanezumab). Their phase II [13–18] and phase III [19–33] trials against placebo have demonstrated the excellent safety and efficacy profile in migraine prevention [39]. Furthermore, several meta-analyzes have supported these results [34–39]. The conclusions that can be drawn from all these studies are that there appear to be no significant differences in safety between each of the CGRPs, and that all of them are superior in efficacy to placebo.

Erenumab was the first CGRP mAb approved for migraine prevention in Europe. The European Medicines Agency approval was communicated the 26^th^ of July 2018 for patients with at least 4 MMDs for the last three months. The Spanish Medicines Agency authorized a personalized managed access program that allowed neurologists to treat patients before the official reimbursement in January 2019. In the same date, the Headache Study Group of the Spanish Neurological Society (GECSEN) started MAB-MIG. This is a prospective, independent, and multicentre registry of migraine patients treated with CGRP mAbs promoted by GECSEN, created to evaluate their real-world effectiveness and tolerability by inviting headache specialists around the country. Here, we present the data of effectiveness and tolerability of the first 210 included migraine Spanish patients after 12 weeks treatment with erenumab.

## Methods

The MAB-MIG scientific committee is constituted by the members of GECSEN board (R. Belvís, S. Santos, G. Latorre and C. Gonzalez-Oria) plus two independent advisors (P. Pozo-Rosich and R. Leira). This committee selected the variables and advised on the design of the database. It also resolved queries of the investigators and assessed the final database and the statistical analyses. Each investigator acted according to their clinical criteria, considering the European Medicines Agency and the Spanish Neurology Society guidelines [3] that establish the indication of erenumab from at least 4 MMDs. The recommendations on erenumab treatment in migraine, proposed by international experts [3, 40–42], were made available to researchers.

All patients included fulfilled the migraine criteria of the International Headache Society (IHS) [43]. Patients were between 18 and 65 years old, had ≥ 4 MMDs for the last three months and were treated with erenumab during a minimum 12-week period. Migraine started in their lives before age 50 and all of them had the migraine diagnosis for a minimum of one year prior to inclusion in the registry. Patients with recent cardiovascular or cerebrovascular events (in the previous three months) were excluded.

We collected the following variables:

1. Demographical data: gender and age.
2. Clinical data as migraine form (with/without aura), MMD and MHD (number of mean monthly headache days). According to these definitions, we considered CM (≥ 15 MHDs) versus episodic migraine-EM (< 15 MHDs). Moreover, the EM group was subdivided into HFEM (10 to 14 MHDs) and low-frequency episodic migraine (LFEM; < 10 MHDs).
3. Effectiveness variables. The following variables were collected at baseline and after 12-weeks treatment with erenumab: number of MMDs and MHDs, and patient-reported outcomes (PROs), including headache impact test (HIT-6) score and the migraine disability assessment questionnaire (MIDAS) score. Finally, patients implemented a patient global impact changes (PGIC) scale to evaluate their satisfaction.

According to the IHS guidelines of controlled trials in migraine [4], *number of MMDs* was considered the primary endpoint. Response was considered when a reduction in the number of migraine days > 50% was observed between baseline and week 12 of treatment with erenumab.

Additionally, we collected other variables: prior preventives drugs taken, including BoNT/A, previous overuse of acute medication, erenumab treatment alone or in combination with another preventive drug, initial erenumab doses, and if there was a change in the erenumab dosage after 12 weeks. Other changes measured were conversion from CM to EM, and medication overuse headache (MOH).

### Tolerability analyses

We collected all adverse events (AEs), and the MAB-MIG scientific committee classified them as related or non-related to erenumab treatment. According to Good Clinical Practice guidelines, we classified adverse events as mild, moderate, or severe, and we collected the dropout rate.

For statistical analysis we used the SPSS software (version 22.0; SPSS Inc., Chicago, IL, USA). Results were expressed as means and standard deviations or as absolute number and percentages. Patient data were classified into two groups: baseline visit and 12-week visit. Comparisons have been made using the Student's t-test for quantitative variables and contingency tables and the chi-square test for categorical variables. When the distribution of the data went out of normality, we used the Mann–Whitney U test. Statistical significance was considered when *p* < 0.05.

Finally, MAB-MIG was classified as a *low-intervention clinical trial* by the Spanish Medicines Agency and was approved by the Ethics Committee of Investigation with Medicines of the Health Area of Valladolid (PI 20–1790). The name of the participant hospitals was anonymized and the information regarding their patients was sent in encrypted form.

## Results

We included 210 patients from 22 Spanish hospitals, from February 2019 to June 2020, who had completed at least 12 weeks of erenumab treatment. The included centres had a homogeneous geographic distribution around the country. The mean age was 46.4 years-old (18–65), and 86.7% of patients were women.

The mean migraine duration was 26.5 years (3–25 years). Most patients (89.5%) had CM with an average evolution of 8.6 years (3 months-25 years) and the remaining presented HFEM (10.5%). Seventy percent of patients presented MOH, and 17.1% fulfilled migraine with aura criteria. The average of MMDs was 17.1 days (4–30), and of MHDs was 23.5 days. The mean MIDAS score was 101.9 points, and the mean HIT-6 score was 68.8 points.

Patients had failed a mean of 7.8 (2–20) preventive treatments at baseline including BoNT/A. The later had been used by 95.2% of patients. The most frequently used oral preventive drugs were topiramate (98.2%), amitriptyline (98.2%), flunarizine (94.7%) and beta-blockers (92.9%).

The initial dose of erenumab was 70 mg in 67.6% of patients and 140 mg in the remaining 32.4%. Regarding simultaneous preventive treatments, only 39.5% patients received exclusively erenumab as preventive treatment**,** and in the remaining patients (60.5%) erenumab was added to another preventive drug that the patient already took. Thus, 27.6% of patients received BoNT/A plus erenumab, 12,2% topiramate plus erenumab and 49.1% a miscellanea of oral preventive drugs plus erenumab.

Regarding effectiveness (Table 1), the responder rate was 37.1%, and the mean reduction in MMDs was 6.5 days (from 17.1 to 11 days). MHDs were also reduced in 8.6 days (from 23.5 to 14.9 days).

**Table 1:** Clinical responses and patient-reported outcomes (PROs) at the baseline period and after week 12 of erenumab treatment

Variable	Baseline	Week 12	Difference
MMDs	17.1 days	11.0 days	-6.5 days
MHDs	23.5 days	14.9 days	-8.6 days
HIT-6 score	68.8 points	57.2 points	-11.6 points
MIDAS score	101.9 points	66.9 points	-35 points
MOH	70%	43.4%	-26.6%

**Fig. 1:**
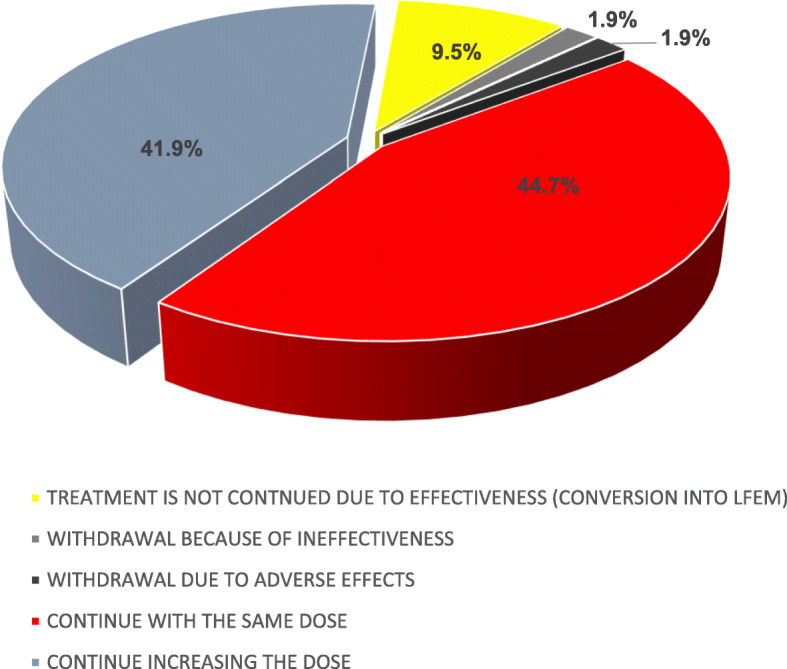
Sector graph showing clustering of the 210 migraine patients according to erenumab response after 12 weeks of treatment

After the 12-week period of treatment (Fig. 1), 28 patients (13.3%) discontinued the treatment. The reasons were: 1) excellent effectiveness that allowed to achieve the conversion to LFEM (20 patients; 9.5%), 2) lack of effectiveness (4 patients; 1.9%), and 3) AEs (4 patients; 1.9%).

The remaining 182 patients (86.7%) continued with erenumab treatment: with the same dose (44.7%), while 41,9% increased the dose thereafter (Fig. 1). After three months of follow-up, 14.8% continued to receive simultaneously BoNT/A and 50% were still under treatment with oral preventive drugs. We want remark that 69 patients (32.8%) continued erenumab despite of they achieved to convert CM into HFEM.

Regarding PROs (Table 1): MIDAS score was reduced 35 points (from 101.9 to 66.9), HIT-6 was reduced 11.6 points (from 68.8 to 57.2) and the mean PIGC assessment was 4.7 points.

We also tried to identify predictive response factors. In this way we found a cut-off point in 5.9 previous preventives failures (*p* = 0.026) (Table 2) above which only the 10% of patients responded to erenumab treatment. Other predictive factors were MIDAS score < 100 points (*p* = 0.006), < 80 points in HIT-6 score (*p* = 0.01), and absence of MOH (*p* = 0.039). All the responder patients showed a HIT-6 score < 80 points making this index in a strong predictor factor of response at this cut point.

**Table 2:** Predictive factors of good response

Variable	Responders (*n* = 78)	Non-responders (*n* = 130)	*p*
Age (years)	47.4 y	45.9 y	0.557
Gender (women) (*n* = 180)	65 (36.1%)	115 (63.9%)	0.294
Aura (*n* = 36)	18 (50%)	18 (50%)	0.088
CM (*n* = 186)	69 (37.1%)	117 (62.9%)	0.727
EM (*n* = 22)	9 (40.9%)	13 (59.1%)	0.907
MOH (*n* = 145)	61 (42.1%)	84 (57.9%)	0.039
Prior BoNT/A (*n* = 198)	74 (37.4%)	124 (62.6%)	0.867
Erenumab 70 mg (*n* = 140)	51 (36.4%)	89 (63.6%)	0.760
Erenumab 140 mg (*n* = 68)	27 (39.7%)	41 (60.3%)	0.648
Simultaneous BoNT/A (*n* = 57)	34 (59.6%)	23 (40.4%)	< 0.001
Simultaneous oral preventives (*n* = 126)	43 (34.1%)	83 (65.9%)	0.213

None of the erenumab doses, 70 or 140 mg, showed a better statistical power as predictor of good response than the other (*p* = 0.647). However, the simultaneous BoNT/A treatment showed the strongest predictor power of a good response (*p* < 0.001). On the contrary, simultaneous oral preventives did not predict the response (*p* = 0.213).

In addition, the presence of aura showed a non-significant tendency as a predictor factor of good response (*p* = 0.088). However, age (*p* = 0.557), gender (*p* = 0.294), the form of migraine HFEM/CM (*p* = 0.727), and evolution of CM (*p* = 0.514) did not show any association to response.

Finally, regarding tolerability, the percentage of AEs was 20%, but only four patients (1.9%), suffered severe adverse events leading to treatment discontinuation. Two patients had a skin rash attributed to the first erenumab injection; the other two patients presented AEs not related to erenumab: one patient, under paroxetine treatment, presented a serotoninergic syndrome while overusing zolmitriptan; and the other one was diagnosed of cutaneous melanoma, but the skin lesion existed previously to the erenumab treatment onset.

Specifically, forty-two patients presented 57 AEs, being constipation the most frequent (7.6%). No patients needed treatment or consultation by this AEs. Table 3 details AEs reported by patients after 12 weeks of treatment with erenumab.

**Table 3:** Adverse events (AEs) collected during the 12 weeks of erenumab therapy

AE	Absolute number and percentage
Constipation	16 (7.6%)
Flu-like symptoms	8 (3.8%)
Pruritus after injection	6 (2.8%)
Fatigue	5 (2.3%)
Dizziness	3 (1.4%)
Nausea after injection	3 (1.4%)
Upper respiratory tract infection	2 (0.9%)
Skin rash after injection	2 (0.9%)
Lymphadenopathy	1 (0.4%)
Serotoninergic syndrome	1 (0.4%)
Loss of sexual desire	1 (0.4%)
Melanoma	1 (0.4%)
Diarrhoea	1 (0.4%)
Myalgia	1 (0.4%)
Injection site pain	1 (0.4%)
Muscular spam	1 (0.4%)
Panic attack	1 (0.4%)
Palpitations	1 (0.4%)
Dehydration	1 (0.4%)
Hypermenorrhoea	1 (0.4%)
Death	0 (0%)
Total	57

Finally, we did not find any predictive factor of AEs, but the dose of 140 mg showed a non-significant tendency to present more AEs than the dose of 70 mg (*p* = 0.069).

## Discussion

We present the first multicentre and prospective real-world experience of erenumab in the preventive treatment of migraine in Spain. Erenumab presents an excellent tolerability profile in our registry, but a slightly lower effectiveness, response rate of 37%, comparing to 39–50% that is the average of phase III clinical trials [19–32], open-label extension studies [44–46], and meta-analysis [34–39].

This can be attributed to the fact that most of the 210 patients included were highly refractory CM patients and therefore would have been excluded from clinical trials [19–32]. For example, the LIBERTY trial [23] analysed erenumab versus placebo in 246 patients with EM who were unsuccessfully treated (in terms of efficacy or tolerability, or both) with 2–4 preventive treatments. The erenumab response rate reported was 50% [23]. Our response rate is lower, but it must be considered that our patients were more refractory (they failed an average of 7 previous preventives), the majority had CM and part of the study was carried out during the most serious phase of the COVID-pandemic.

Another explanation could be that the more frequent erenumab initial dose prescribed was 70 mg because initial dose was a discretionary decision of the investigator. Since patients included in our study have a long history of migraine, a high number of MMDs, numerous failures to preventive migraine drugs and high impact in HIT-6 and MIDAS scales compared with patients from clinical trials, perhaps effectiveness could have been better if all the investigators had started the treatment with the 140 mg dose.

As expected, the lower the scores in the HIT-6 and MIDAS scales, and the fewer the number of preventive drugs that have previously failed, the more likely the erenumab treatment will be effective. These are the effectiveness predictors that we have found in our study, together with the absence of MOH. Nevertheless, one unexpected predictive factor in our study was the simultaneous treatment with erenumab plus BoNT/A. This association was the strongest predictive factor of a good response and showed an excellent tolerability profile.

A huge number of real-world experiences analysing erenumab in migraine prevention are being published around the world [47–63]. These experiences already include more than 2,000 patients with migraine, and, among them, we can find eleven one-centre studies (seven prospective [48, 53, 54, 56, 57, 59, 61] and four retrospectives [50–52, 62]) and five multicentre (four prospective [49, 55, 58, 60] and one retrospective [63]). Our registry includes the second largest sample of migraine patients treated with erenumab in a multicentric prospective registry. A published Italian study [55] included more patients, 372 patients, but this initiative was composed by a group of headache experts and it included only ten Italian centres, and nine of them were localized in the north of Italy. Our study is the official registry of the Spanish Neurological Society and includes 22 centres with homogeneous representation of the country. Moreover, the average number of prior preventive drug failures was 3–5 in the Italian study [55] and superior to 7 in ours, which means that our patients are more complex and treatment-refractory than the patients of the Italian study. Despite these differences, both the Italian study [55] and the other real-world experiences [47–63] conclude, like our registry, that erenumab is useful in the prevention of EM and CM and presents a good tolerability profile.

Erenumab has shown scarce adverse events in our registry (20%), like in the phase II [13–18] and phase III [19–32] clinical trials, meta-analysis [34–39], open-label extension studies [44–46] and real-world experiences [46–62]. Most of the adverse events were mild and transient in our study. Mild constipation, flu-like symptoms, transient pruritus at the injection site and fatigue were the only adverse events with incidences superior to 2%. We only collected two severe adverse events related to erenumab treatment (two skin rash after injection) that represent 0.9% of our patients, a similar figure to that of clinical trials and real-world experiences (1–3%) [19–39, 44–62].

Regarding the initial dose of erenumab, we have not found any difference on effectiveness between them, unlike other studies [64]. On the other hand, the excellent tolerability pattern of the two doses of erenumab is already known and our study confirms it, despite the 140 mg dose showing a non-significant trend to be related to more adverse events than the dose of 70 mg.

This first report of the results of the MAB-MIG registry has some limitations. First: patients included are the most refractory ones of Spanish headache units and they were waiting the arrival of mAbs. For this reason, they do not exactly represent the Spanish real-world experience. Second: we present effectiveness and tolerability results at three months of therapy, a short follow-up. Finally, we have not analysed the comorbidities existence. Despite these limitations, MAB-MIG results have great strength because they are the first post-marketing results of erenumab collected by a neurology scientific society in 22 hospitals in one European country.

## Conclusions

Our registry supports the tolerability and effectiveness of erenumab in the real-world clinical setting. In this way, one out three highly refractory migraine patients responded to erenumab with almost no relevant side effects.

Likewise, a high number of failures with previous preventive drugs, overuse of symptomatic medication and high degrees of disability are the main predictors of poor response. On the other hand, the concomitant use of BoNT/A plus erenumab seems to present an excellent tolerability profile, as it has already been proposed in several studies [65, 66] and is the strongest predictive factor of good response.

## Data Availability

Generated data in the MAB-MIG registry are not publicly available due to the Spanish law for the protection of personal data but are available from the corresponding author on reasonable request.

## Abbreviations

BoNT/A: Botulinum toxin type A
CGRP: Calcitonin gene-related peptide
CM: Chronic migraine
EM: Episodic migraine
HFEM: High frequency episodic migraine
HIT-6: Headache impact test
ICHD: International Classification of Headache Disorders
IHS: International Headache Society
LFEM: Low frequency episodic migraine
mAbs: Monoclonal antibodies
MHD: Monthly headache days
MIDAS: Migraine disability assessment questionnaire
MMD: Monthly migraine days
MOH: Medication overuse headache
PGIC: Patient global improvement change
PROs: Patient-reported outcomes
